# Knowledge, perceptions, practices, promotive factors, and health risks awareness of African Basotho women towards skin lightening products: a cross-sectional survey

**DOI:** 10.11604/pamj.2023.44.43.27230

**Published:** 2023-01-24

**Authors:** Nthabiseng Florina Motlohi, Eltony Mugomeri, Clemence Tarirai

**Affiliations:** 1 Department of Health Information Management, Faculty of Health and Education, Botho University, Maseru, Lesotho,; 2 Department of Health Sciences, College of Agriculture and Natural Sciences (CHANS), Africa University, Mutare, Zimbabwe,; 3 Department of Pharmaceutical Sciences, Faculty of Science, Tshwane University of Technology, Arcadia, Pretoria, South Africa

**Keywords:** Women, skin lightening products, knowledge, health risks awareness, cross-sectional survey

## Abstract

**Introduction:**

the use of skin lightening products (SLPs) by women is poorly documented in Africa, with statistics from some countries entirely missing. This study assessed knowledge, perceptions, practices and factors associated with health risk awareness of African Basotho women towards SLPs.

**Methods:**

this was a questionnaire-based cross-sectional study based on convenience sampling of females in secondary/high schools, universities, factories and business offices in Maseru City, Lesotho. Analysis of the differences in knowledge (adequate ≥50% score), perceptions, and practices between four participant groups was based on ANOVA, p<0.05. Associations between sociodemographic variables and the use of SLPs were performed using logistic regression model in SPSS version 27.

**Results:**

a total of 468 participants out of 496 responders qualified for data analysis based on predefined data cleaning criteria. Knowledge about SLPs was adequate (78.2%, n=468). By proportion, the main sources of the SLPs were supermarkets (67.6%, n=183) and pharmacy stores (41.9%). About 43.7% (n=468) of the participants used SLPs, with the factory workers mostly associated with SLPs use (aOR: 2.91, 95% CI 1.15-7.40; p=0.02). The majority (53.4%, n=131) of users had inadequate knowledge about the link between skin lightening and skin problems. The most common reasons for use of SLPs were rash (pimples, blemishes) (43.9%, n=107), dry skin (41.1%) and skin reddening (33.6%).

**Conclusion:**

there was adequate knowledge and moderate practice of skin lightening among African Basotho women. Public awareness campaigns and strict regulations are required to address the problem of SLPs use.

## Introduction

Skin lightening is a cosmetic application of topical ointments, creams or emulsions, gels or lotions and soaps, which contain chemicals that inhibit secretion of melanin, a brown/black colored pigment that protects the human skin from damage by sun rays [1]. Chemicals that are often used include mercury, corticosteroids, and high quantities of hydroquinone [2]. Mercury is highly toxic, and its prolonged use may lead to neurological disorders and kidney malfunction [3]. Hydroquinone, on the other hand, is a potent inhibitor of melanin [4]. Exposure to the sun will easily damage skin treated with these chemicals [5]. Hydroquinone is banned in Japan, the European Union and Australia because of its toxicity [6]. Skin lightening products have been used for applications ranging from clinical to cosmetic reasons in many parts of the world for centuries despite the adverse effects [7]. Most SLPs are used in the treatment of hyperpigmentation disorders such as melasma and post-inflammatory hyperpigmentation [8,9]. However, SLPs have been used off-label for general cosmetic skin lightening [1,10-13]. Skin lightening products are used mostly on the face and hands, with whole body application being popular as well [14]. Common adverse effects to SLPs include less systemic effects such as acne, skin redness, contact eczema and sunburn possibly due to skin thinning (epidermal atrophy) [2,12,15]. More serious effects include poor wound healing, visible blood vessels, exogenous ochronosis (bluish black tissue discoloration), skin cancer, generalized kidney dysfunction and Cushing´s syndrome [2,12,15]. Furthermore, bacterial and fungal infections have also been associated with the use of topical corticosteroids [2]. More clinical evidence is required to establish the association between SLPs and these potential side effects. Although both men and women use SLPs globally, it is more common among women particularly the darker-skinned ones [12,16,17]. In Africa, the use of SLPs ranges between 25% and 77% with Mali reporting 25%, South Africa 32%, Ghana 39%, Senegal 50%, Congo-Brazzaville 66%; while the highest is Nigeria with 77% [1,10-13,15]. Despite the practice being more prevalent in townships and among city women [12], the practice does not differ by educational background. Women with no formal education through to professional women with tertiary education use SLPs [12,15,17]. Although the use of SLPs as cosmetics is common among darker-skinned women of African descent, research on the knowledge, perceptions and use of SLPs among African Basotho women, is lacking [18-20]. The overarching aim of the study was to assess the knowledge, perceptions, practices, factors associated with skin lightening, and health risk awareness of African Basotho women towards SLPs.

## Methods

**Study setting:** Lesotho is a small African country landlocked and enclosed entirely within South Africa. The population is around 2 million with 51% females and 49% males [21,22]. The country is divided into 10 districts with Maseru as the capital city. About 25% of the population lives in the capital city. The female population in Maseru is estimated at 255 000 women with 61% of these being in the 15-64 years age group and 62% living in urban areas [21,22]. The national literacy rate was estimated at 97.0% in 2016 among Basotho women [23,24]. In Lesotho, currently there are no SLPs policies and regulations.

**Study design:** a cross-sectional study was carried out to assess knowledge, perceptions, practices and factors associated with skin lightening among African Basotho women in Lesotho. A self-administered questionnaire was employed, which consisted of closed- and open-ended questions categorized into eight sections, such as participants´ sociodemographic information including gender, level of education, nationality, marital status, occupation; knowledge of SLPs, sources of SLPs, uses, reasons for use, and perceptions about SLPs.

**Study population and sampling:** the female population in Maseru City is estimated at 255, 000 women with 61% of these in the 15-64 years age group, which constituted approximately 155,550 women in the overall study population [21,22]. The study targeted Basotho females of African descent aged 18-60 years in four social groups, namely; a) secondary/high school pupils; b) university students; c) textile factory workers; and d) professional women, in Maseru. Males, non-Basotho and non-African Basotho women, and African Basotho females younger than 18 years or older than 60 years were excluded from the study. A modified convenience sampling approach independent of the researchers´ control was adopted. The study was limited to the consenting age group of 18-60 years for accessibility reasons. Additionally, since Lesotho´s total population consists of 99% African Basotho and 1% other nationals [22], non-Basotho African women were excluded from the study. For sampling, at least 200 questionnaires in each of the four social groups were distributed with the help of research assistants. Participants were approached during break or lunchtime. Overall, 468 participants responded with usable questionnaires.

**Data collection and capturing:** the questionnaire was designed in English and translated to native Sesotho by one of the researchers (NFM). The questionnaire was coded for data analysis before data collection. The questionnaire was pilot tested with 20 participants around Maseru City and updated accordingly. Data collection was conducted between October and December 2019. After data collection, questionnaires were checked for completeness and only those with 75% completeness or more were captured and analyzed. Notably, participants who had never used the SLPs and could only respond to sociodemographic and knowledge of skin lightening questions were excluded from perceptions and practices during data analysis.

**Data analysis:** sociodemographic data was analyzed using frequencies and percentages and presented in a tabular form. The association between sociodemographic (independent variables) and use of SLPs (dependent variable) was analyzed using univariable and multivariable binary logistic regression model with confidence interval (CI) at 95%, and p≤0.05. Independent variables that showed a significant (p≤0.05) association with the use of skin lightening products were subjected to multivariate analysis. Knowledge was regarded as adequate if a participant scored ≥50% in a pre-defined cluster of knowledge. The use of SLPs was regarded as low (<40%), moderate (40%-50%), or high (>50%) based on the proportion of participants who have used SLPs. Furthermore, differences between participant sociodemographic groups were assessed using one-way ANOVA (p≤0.05). Statistical analyses were performed using IBM Statistical Package for Social Sciences (SPSS) version 27.

**Ethical considerations:** ethical approval was granted by Botho University (Ethics reference no: 12/OQM/GBE/2019) and the Lesotho Ministry of Health (Ethics reference no: ID144-2018). Permission to conduct the study at the study sites was granted by high school principals, university rectors, and company or industrial/factory managers. Written informed consent was obtained from each study participant.

## Results

**Sociodemographic characteristics of the respondents:** a total of 468 participants out of 496 responders qualified for data analysis. A sample size of 468 participants was therefore realized for analyses, which was 117% (n=400) of the minimum target sample. Of the 468 participants, the main participants were university students (35.5%), industrial/factory workers (34.2%), with the least group being professional women (18.8%) and secondary/high school learners (11.5%). Table 1 summarizes the sociodemographic characteristics of the participants. There was statistically significant difference (p<0.05) among sociodemographic subgroups of age group, occupation and economic status. The most common age group was the young adult category aged 21-25 years (23.2%, n=465), followed by 18-20 years (21.5%), with the least common age group being the elderly in the 56-60 years category (1.1%). The study revealed that 58.3% had tertiary education while 32.1% had secondary/high school and 8.1% had primary education, respectively. Only 1.5% of the total participants did not have any formal education. About 51.7%, (n = 466), of the participants were single, 36.9% married, 6.2% widowed, 4.7% were divorced while 0.5% were cohabiting. The majority ((64.3%) (n=428)) of participants could eke out a living while 35.7% struggled to make a living.

**Table 1:** socio-demographic characteristics of the participants

Variable	Subcategory	Frequency	Percentage (%)	p value
**Age group (years) (n=465)**	18≤20	100	21.5	0.002*
	21≤25	108	23.2	
	26≤30	69	14.8	
	31≤35	69	14.8	
	36≤40	53	11.4	
	41≤45	23	4.9	
	46≤50	24	5.2	
	51≤55	14	3.1	
	56≤60	5	1.1	
**Current occupation/activity (n=468)**	Secondary/high school	54	11.5	0.003*
	learner	166	35.5	
	University student	160	34.2	
	Factory worker	88	18.8	
	Professional women			
**Education (n=468)**	No formal education	7	1.5	0.153
	Primary education	38	8.1	
	Secondary education	150	32.1	
	Tertiary education	273	58.3	
**Marital status (n=466)**	Married	172	36.9	0.168
	Divorced	22	4.7	
	Widowed	29	6.2	
	Single	241	51.7	
	Co-habiting	2	0.5	
**Economic status (n=428)**	Sufficiently get by	275	64.3	0.017*
	Struggle to get by	153	35.7	
**Location (n=463)**	Urban or city suburbs	380	82.1	0.896
	Rural or township	83	17.9	

* = Statistically significant difference

**Knowledge, practices, perceptions, and health risk awareness towards SLPs:** knowledge, practices, perceptions, and health risk awareness of African Basotho women towards SLPs are presented in Table 2. Most participants (78.2%, n=468), had adequate knowledge about SLPs with 43.6% of these reporting they have used the products. Sources of information about SLPs were widely spread among the participants. Most participants learned about SLPs from friends (49.2%, n=356) and family members (25.6%) with partners being the least source of SLPs information. Most users purchased SLPs from supermarkets (67.4%, n=138) and pharmacies (41.9%). An online purchase was the least preferred source (4.3%). Methods of application of these products on the body differed among users. Most participants applied the products on the entire face (66.2%, n=142) while 19.7% applied the products on the entire body. Application of SLPs on problem surfaces (14.1%) was also reported. The study revealed that 11 different products were used as SLPs. These products contained corticosteroids, hydroquinone, and mercury according to information provided by the participants. Most participants used SLPs to enhance beauty (48.8%, n=129) and to treat skin problems (47.3%). About 11.6% reported the reason for use as peer pressure. Although some women used SLPs to treat skin problems, these products could result in other skin problems mainly rash (43.9%, n=107) and dry skin (41.1%).

**Table 2:** knowledge, perceptions, practices, and health risk awareness of African Basotho women towards skin lightening products (SLPs)

Variable	Subcategory	Frequency	Percentage (%)
**Knowledge about SLPs (n=468)**	Yes	366	78.2
	No	102	21.8
**Use of skin lightening product (n=359)**	Yes	157	43.7
	No	202	56.3
**Source of product information (n=356)**	School	76	21.3
	Clinic	44	12.4
	Family member	91	25.6
	Partner	16	4.5
	Friends	175	49.2
	Publications(e.g. magazines)	85	23.9
	Internet	84	12.9
	Radio or tV	64	9.8
	Other	15	2.3
**Product application (n=144)**	On the entire face	94	66.2
	On the whole day	28	19.7
	On problem areas	20	14.1
**Period of use (n=136)**	Less than a year	71	52.2
	A year or more	65	47.8
**Point of purchase**	Supermarket	93	67.6
**(n=138)**	Pharmacy/chemist	57	41.9
	Street vendor	26	18.4
	Online	6	4.4
	Other	1	0.7
**Cost (n=129)**	M50	110	85.3
	M50 – M100	12	9.3
	Above M100	7	5.4
**Reasons for use (n=129)**	Beauty enhancement	63	48.8
	Skin problems	61	47.3
	Peer-pressure	15	11.6
	Other	1	0.8
**Awareness of the side effects associated With SLPs use (n=131)**	Yes	61	46.6
	No	70	53.4
**Occurrence of side effects (n=107)**	Skin reddening	36	33.6
	Skin discoloration	27	25.2
	Rash/pimples	47	43.9
	Dry skin	44	41.1
	Dark complexion than normal	25	23.4
	Skin itching	28	26.2
	Other	4	3.7
**Use of sunscreen (n=127)**	Yes	35	28.0
	No	92	72.0
**Product satisfaction (n=91)**	Yes	36	39.6
	No	55	60.4

**Figure 1:**
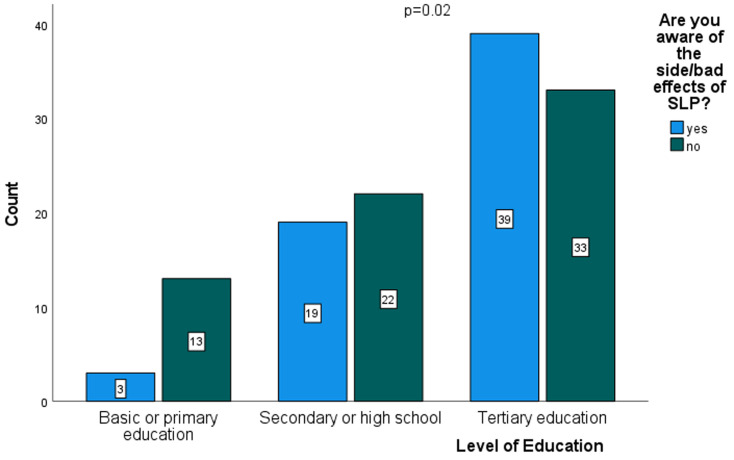
awareness of health problems associated with the use of skin-lightening products stratified by level of education

**Factors associated with the use of skin lightening products and health awareness risks of African Basotho women:** the results of a binary logistic regression are presented in Table 3. Basotho's women aged below 30 years were less likely to use SLPs than older women (OR: 0.27, 95% CI 0.09-0.83; P=0.02). Similarly, women who did not struggle economically were less likely to use SLPs than those who perceived themselves as economically struggling (OR: O.60, 95% CI 0.38-0.96, P=0.03). Contrarily, univariate analysis on the use of SLPs using current occupation as the predictor showed that women were 1.81 more likely to use SLPs if they were working in the factories than if they were professional women (OR: 1.81, 95% CI 1.00-3.27; P=0.05). Additionally, there was a higher likelihood of using SLPs among divorcees and widowers than in single women (OR: 2.37, 95% CI 1.18-4.74; p=0.02). After controlling for confounders, only current occupation showed a significant difference in SLPs use between professional women and factory workers (aOR: 2.91, 95% CI, p=0.02), which implies that factory women workers were 2.91 more likely to use SLPs than professional women. Figure 1 shows a comparative analysis for awareness of health risk associated with SLPs use between participants with different levels of education. Women with primary education or less were significantly (p=0.02) less likely to be aware of the health risks associated with SLPs use than women with higher level of education.

**Table 3:** association between independent variable (SLPs use) and independent predictors

Variable	subcategory	Univariate analysis		Multivariate analysis	
		OR (95% CI)	p value	aOR (95% CI)	p value
**Age group (years)**	≤30				
	31-50	0.27 (0.09-0.83)	0.02*	0.29 (0.07- 1.16)	0.08
	51-60	0.58 (0.19-1.80)	0.34	0.45 (0.12- 1.63)	0.22
**Current occupation**	Secondary/high school learner	0.46 (0.21-1.03)	0.06	0.97 (0.24-3.84)	0.96
	University student	0.86 (0.47-1.57)	0.61	1.10 (0.48-2.50)	0.82
	Factory worker	1.81 (1.00-3.27)	0.05*	2.91 (1.15-7.40)	0.02*
	Professional women				
**Education**	No formal/Primary education	2.41 (1,12-5.16)	0.02*	1.07 (0.36-3.22)	0.90
	Secondary education	0.96 (0.61-1.51)	0.87	0.47 (0.19-1.16)	0.10
	Tertiary education				
**Marital status**	Married	1.32 (0.84-2.07)	0.22	0.78 (0.41-1.48)	0.45
	Once married (divorced/widowed) Single (single/co-habiting)	2.37 (1.18-4.76)	0.02*	1.02 (0.39-2.69)	0.96
**Economic status**	Sufficiently get by	0.60 (0.38-0.96)	0.03*	0.69 (0.40-1.17)	0.17
	Struggle to get by				
**Location**	Urban or city suburbs	0.98 (0.55-1.76)	0.95	1.29 (0.64-2.57)	0.48
	Rural or township				

OR = Odds ratio, aOR = adjusted Odds ratio, CI = Confidence interval. * = Statistically significant difference

## Discussion

The results revealed that the majority of the participants had adequate knowledge about SLPs while nearly half of the participants moderately used the SLPs. This implies that awareness was not synonymous with usage and that a significant proportion of users of the SLPs are using them regardless of their knowledge of the dangers associated with the use of SLPs. Importantly, there is a need to establish the reasons for this behavior. Possibly, to a significant proportion of the users of SLPs, the perception that lighter skin is superior forces them to disregard the dangers. More action is therefore needed in addition to awareness campaigns. A relevant policy or regulation to restrict the availability of SLPs is therefore imperative to address this problem. To put the reported usage of SLPs in this study into context, Basotho women are among the least users of SLPs in sub-Saharan Africa (SSA), with women in Ghana being the most comparable at 39% in Ghana [12]. However, the 77% usage rate among Nigerian women [10] mentioned earlier ranks much higher compared to Basotho and Ghanaian women. The reasons behind the differences in SLPs use among African women in different SSA countries need to be explored, including the respective policies and regulations. The use of SLPs among Basotho women was more prevalent in industrial/factory workers and least prevalent in secondary/high school learners (Table 3). According to Kuffour *et al*. (2014), the level of education and use of SLPs are interrelated: a lower level of education is associated with high SLPs use [12]. Most Basotho industrial/factory workers have lower education, which is comparable to secondary/high school learners. Industrial/factory workers most likely used SLPs because they are employed and can afford the products since 85.0% of the products' costs lower than M50.00 (Table 2), unlike the learners who are dependent on their parents for financial support. Women with a tertiary level of education were aware of health problems associated with SLPs use compared to women with primary and secondary/high school education or women with no formal education. This relationship better explains the low uptake or use of SLPs observed among professional women with tertiary level education compared to a higher uptake among industrial/factory workers who have mostly primary and secondary/high school education. It further underscores the interrelationship between education and the use of SLPs [11,12]. Public health awareness measures on SLPs should target young women as early as secondary/high schools for primary prevention of skin lightening practices. The reasons for the use of SLPs among Basotho women were mainly beauty enhancement and treatment of skin conditions at similar prevalence proportions. However, more SLPs-related skin conditions can be predicted in the future since the majority of the respondents reported skin problems, mainly skin rashes. Furthermore, the low usage rate of sunscreen by Basotho women who used SLPs underpins the need for awareness campaigns for SLPs providers including health practitioners to ensure that patients understand the risks of SLPs and the importance of the use of sun screening prior to the initiation of treatment. A concerted effort by dermatologists and other healthcare workers is critical in addressing this matter.

This study also revealed that clinics are an important source of SLPs for Basotho women. It is possible that some of the SLPs are medically prescribed at clinics and the respondents continue to use them beyond the treatment period after the realization that they lighten up the skin and successfully treated the skin conditions. Therefore, users opt for alternative points of purchase such as supermarkets, pharmacies, and street vendors (Table 2). This calls for policymakers to tighten regulatory measures for the sale of SLPs and provide guidelines for the control of the concentration of active ingredients that can be purchased outside of health care facilities. For example, the sale of hydroquinone is banned, or strict control is imposed on its sale in other parts of the world including in South Africa [6,11]. This measure resulted in reduction in the prevalence of skin conditions in South Africa [11]. In addition, the advertisement for SLPs on media platforms such as newspapers, magazines, radio, and television should be regulated to encourage the sale of safe SLPs. These findings of this study should be interpreted with caution since the study has some limitations. A high number of participants completed the sociodemographic section, but did not answer questions seeking knowledge, practice and perception on SLPs hence were not included in data analysis. Limited time could be one of the factors that led to incomplete questionnaires especially among the industrial/factory workers. They hardly had an hour for lunch which was the time required to complete the survey, and were in a hurry to return to work. The study excluded the ages <18 years, which required parental consent, but could potentially have contributed a plethora of information about the skin lightening practices among teenage African Basotho females compared with their seniors. Nonetheless, the study has shed light on the knowledge, practices, and awareness of health risks associated with the use of SLPs. The findings will guide future more research designs.

## Conclusion

There was adequate knowledge and moderate practice of skin lightening among African Basotho women. Public awareness campaigns and strict regulations are required to address the problem of SLPs. Educational campaigns targeting different social groups of women are necessary to raise awareness about health implications of skin lightening and hazardous substances contained in the products. These campaigns require a joint effort from community groups to health workforce in pharmacies and clinics. Legislation that governs the quality of cosmetic products is required in Lesotho. Cosmetics that contain harmful substances such as mercury, hydroquinone and corticosteroids should be banned.

### What is known about this topic

- *Skin lightening products have been used for applications ranging from clinical to cosmetic reasons in many parts of the world;*
- *Skin lightening products use is more common among women particularly the darker-skinned ones;*
- *Comparison of the use of skin lightening products with occupation among African Basotho women*.

### What this study adds

- *Practice of skin lightening products use among African Basotho women;*
- *Prevalence and associated health risks of skin lightening products use among African Basotho women;*
- *Knowledge of skin lightening products associated health risks by level of education among African Basotho women; comparison of the use of skin lightening products with occupation among African Basotho women*.
